# Selective pressure: Rise of the nonencapsulated pneumococcus

**DOI:** 10.1371/journal.ppat.1007911

**Published:** 2019-08-29

**Authors:** Jessica L. Bradshaw, Larry S. McDaniel

**Affiliations:** Department of Microbiology and Immunology, University of Mississippi Medical Center, Jackson, Mississippi, United States of America; Tufts Univ School of Medicine, UNITED STATES

## Introduction

*Streptococcus pneumoniae* (pneumococcus) remains the leading cause of bacterial otitis media (OM), pneumonia, and meningitis despite routine vaccination spanning decades [1,2]. High morbidity, mortality, and financial burdens associated with persistent pneumococcal infections expose the need to reevaluate existing prevention measures [3]. The only currently licensed prevention measures are two vaccines, Pneumovax 23 and Prevnar 13, which target specific polysaccharides that encapsulate the pneumococcus [4]. A major shortcoming of these vaccines is the focus on polysaccharide antigens that are not expressed by the majority of pneumococcal strains. For instance, licensed pneumococcal vaccines collectively cover roughly 25% of known serotypes and elicit no protection against nonencapsulated *S*. *pneumoniae* (NESp). Moreover, nonvaccine serotypes and NESp prevalence have markedly increased since the introduction of pneumococcal vaccines [5,6]. However, the majority of pneumococcal research has focused on encapsulated strains, and there is a large knowledge gap covering the pathogenic potential of emerging NESp. A greater understanding of the risks and diseases associated with NESp is needed to develop broad prevention measures that reduce overall pneumococcal disease. Here, we discuss factors that increase the risk for severe NESp infections and future directions necessary for reducing pneumococcal disease incidence and spread of antimicrobial resistance.

## Nonencapsulated pneumococci are hidden among us

Pneumococci can fail to express capsule if there is a disruption, mutation, or deletion of genes in the capsular polysaccharide biosynthesis (*cps*) locus [7]. Although classically considered avirulent, recent emergence of genetically divergent NESp strains has generated interest in how these bacteria are persisting and evolving [8]. Interestingly, a subgroup of NESp encodes novel virulence-associated proteins in the *cps* locus [7]. These novel proteins compensate for lack of capsule expression by increasing NESp colonization of the host and enhancing virulence during OM and pneumonia in animal models of infection [9–11].

Moreover, small NESp colonies are frequently overlooked, leading to an underestimation in NESp prevalence. In-depth sequencing analysis of pneumococci isolated from human samples has just begun to elucidate NESp-associated diseases. These studies have characterized NESp as efficient colonizers of the human nasopharynx and common causative agents of upper respiratory infections [8,12]. The prevalence of NESp in human carriage isolates is 4% to 19% in geographically distinct regions spanning multiple continents, with higher prevalence found in vaccinated populations [8]. Notably, nearly all conjunctivitis cases are associated with NESp, and NESp strains are isolated from 10% to 15% of OM infections [8]. Because NESp surface proteins are not masked by a capsule, these strains have more intimate interactions with the host cell that permits greater adherence necessary for colonization, which enhances the subsequent risk of developing OM, pneumonia, and bacteremia.

## Ecological perturbations in pneumococcal populations are driving NESp persistence

**Fig 1:**
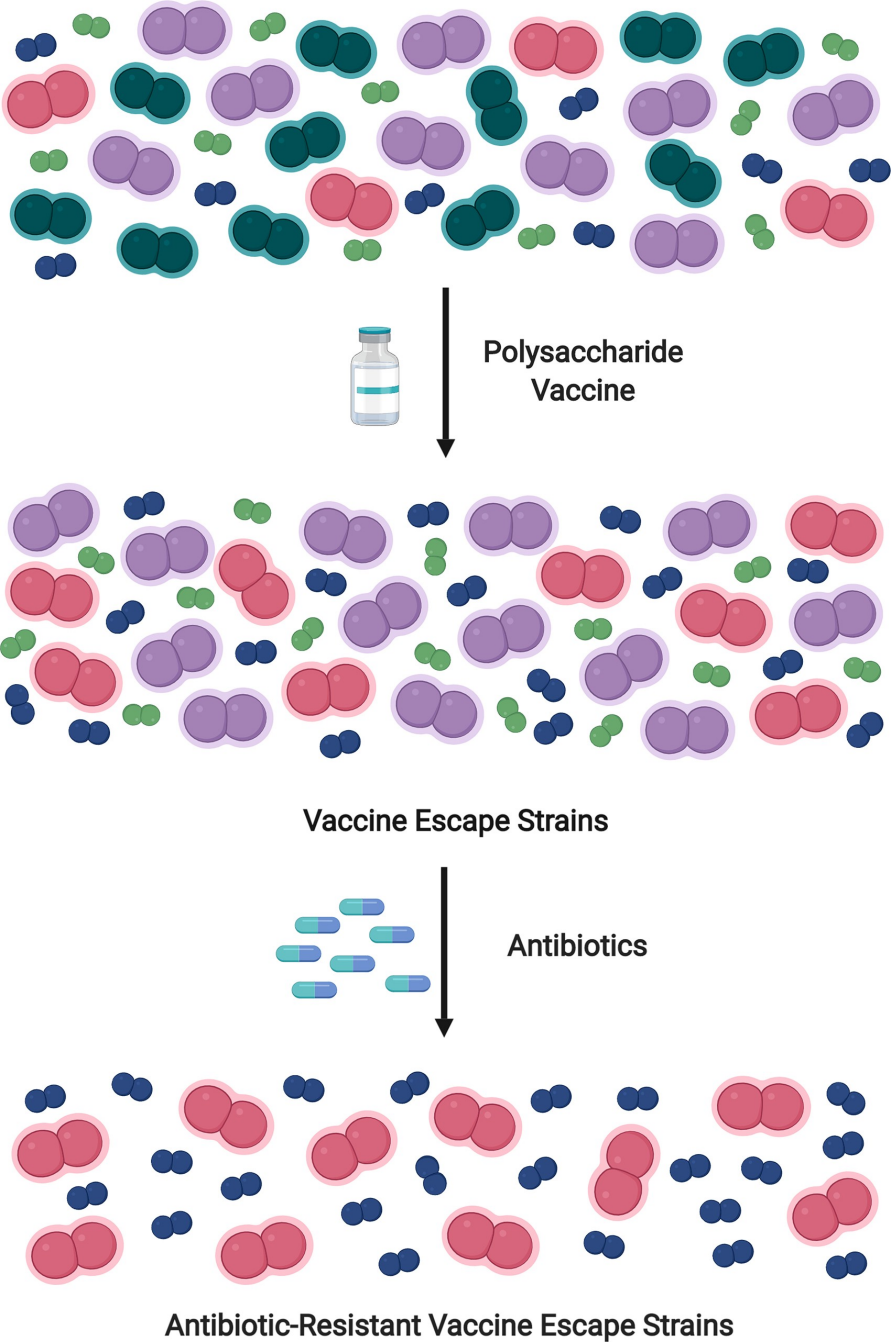
Polysaccharide vaccines and antibiotic use increase risk of NESp infections. Pneumococcal vaccines reduce prevalence of vaccine serotypes (teal). Reductions in vaccine serotypes cause propagation of nonvaccine serotypes (pink and purple) and nonencapsulated pneumococci (blue and green). Antibiotic selective forces drive the outgrowth of antibiotic-resistant NESp (blue) and nonvaccine serotypes (pink). Teal = vaccine serotypes; purple = antibiotic-susceptible nonvaccine serotypes; pink = antibiotic-resistant nonvaccine serotypes; green = antibiotic-susceptible NESp; blue = antibiotic-resistant NESp. NESp, nonencapsulated *S*. *pneumoniae*.

Prevention and treatment measures are selective pressures that drive bacterial population dynamics. Much like a vending machine, “we get what we select” by using limited vaccines or antibiotic pressures that select for vaccine-escape and antibiotic-resistant subpopulations (Fig 1). The pneumococcus is a naturally transformable bacterium that can rapidly take up DNA from its environment and alter phenotypes [13]. This ability to adapt rapidly creates opportunity for pneumococcal persistence and propagation of traits favorable for survival, including antibiotic resistance and host evasion mechanisms. Specifically, highly conserved genes forming a recombination hotspot flank the *cps* locus [14]. This hotspot allows for rapid gene alterations leading to capsule switching or nonencapsulated phenotypes that escape vaccine-mediated antibody responses [15]. Furthermore, large reductions in vaccine serotypes alter the host niche to favor outgrowth of nonvaccine strains. Thus, vaccine-induced pressures on pneumococcal populations have caused a shift in strains associated with disease rather than eradicating pneumococcal disease.

Additionally, NESp are far more efficient at taking up DNA compared with their encapsulated counterparts [16]. Consequently, NESp have high genetic plasticity and serve as antibiotic resistance reservoirs, with 80% to 96% of isolated NESp carrying resistance to multiple antibiotics, including erythromycin, clindamycin, tetracycline, sulfamethoxazole-trimethoprim, and penicillin [8]. Pneumococcal-associated OM is the main reason for pediatricians prescribing antibiotics [17]. Pediatric OM is often recurrent, resulting in periodic exposure to antibiotics. This strong antibiotic selective pressure further favors NESp outgrowth. Overall, the selective pressure of vaccines creates outgrowth of pneumococcal subpopulations, and additional antibiotic selective pressure further permits outgrowth of persistent, antibiotic-resistant NESp (Fig 1).

## Invasive NESp disease: No sugar coating required

Ecological forces that permit the outgrowth of NESp also enhance the risk of NESp-associated invasive disease. Recent surveillance and animal models of infection provide growing evidence that NESp are evolving to persist in invasive environments [8,11,18]. In geographically distinct regions, NESp strains have consistently been isolated during invasive pneumococcal disease (IPD) [18–20]. The majority of these studies characterized IPD samples obtained from children less than 5 years of age, and it is uncertain whether NESp are associated with IPD in adolescents or adults. Without a protective capsule to shield the pneumococcus from antibody and complement factors, NESp must be able to counteract complement deposition in order to evade the host response. Remarkably, NESp encode surface proteins AliC and AliD that reduce complement deposition, mediate evasion of killing by leukocytes, and sequester host Immunoglobulin A (IgA), which is an antibody that is abundant at mucosal surfaces colonized by NESp [11].

Comorbidities may also be contributing factors to NESp-associated invasive disease. Patients with sickle cell disease, chronic obstructive pulmonary disorder (COPD), and human immunodeficiency virus (HIV) and cancer patients undergoing immunosuppression are at an increased risk for developing pneumococcal infections [21]. It is recommended that these patients receive pneumococcal vaccinations or prophylaxis. This unique combination of enhanced opportunity and therapeutic-driven selectivity supports NESp outgrowth and establishment of invasive disease in these patient populations. Altogether, the current risk of NESp-associated invasive disease is minimal, with only 1% to 15% of IPD cases associated with NESp depending on the region, but selective pressures and NESp persistence are slowly establishing a suitable environment for a devastating rise in invasive disease [8].

## Preventing NESp infections: Search for a broad vaccine candidate

The major weaknesses of the currently licensed polysaccharide-based vaccines are poor induction of mucosal immunity and limited efficacy against the majority of pneumococcal strains [4,22]. A strong mucosal immune response is required to protect against middle ear infections and nonbacteremic pneumonia. Licensed pneumococcal vaccines target the disseminated phase of pneumococcal infection rather than mucosal regions where infections persist. Thus, an all-inclusive, protective vaccine candidate must elicit mucosal responses with broad specificity. Due to their immunogenic potential, protein-based vaccines have become increasingly attractive. Major targets of protein-based vaccines are highly conserved, serotype-independent, and immunogenic proteins that greatly contribute to pneumococcal pathogenesis [4,22]. Choline binding adhesion proteins, pneumolysin toxin, secreted proteases, and nutrient-sensing proteins are all attractive vaccine candidates that are conserved between encapsulated and nonencapsulated pneumococci. These vaccine candidates have been tested individually and in combination or as fusion proteins to test immunogenicity and efficacy [4]. Overall, the use of multivalent protein vaccines provides a robust, broad prevention of pneumococcal disease. Yet there has not been a complete shift from including serotype-specific polysaccharide antigens in development of novel vaccines. Rather, some studies incorporate pneumococcal proteins into the currently used polysaccharide conjugate vaccines [4]. Unfortunately, very few of these vaccines are undergoing evaluation in clinical trials. However, whole-cell pneumococcal vaccination using a nonencapsulated strain is undergoing testing in a human clinical trial and has produced promising results in producing strain-independent, protective antibody responses to highly conserved pneumococcal proteins [22]. Yet there is a long road ahead in production and implementation of protein-based pneumococcal vaccines. Until these alternative vaccines are introduced, mucosal and NESp-associated infections will continue to rise and cause severe morbidity and financial burden.

## Combating resistance: Treatment strategies that reduce selective forces

A major risk associated with NESp infections is the propagation of antibiotic resistance [8]. To counteract our dependence on antibiotics and prevent the progression of multidrug resistance, alternative treatment strategies that boost immune responses and decrease host cell damage are necessary. This approach strays away from weakening the bacterium and focuses on strengthening the host, which is an approach that could have expansive applications in comparison to the narrow spectrum of antibiotics. Many pathogens are able to cause disease by disrupting the host immune response. Therefore, therapeutics that boost the host immune response can be used in many infection settings of various origins. Cytokines are small, soluble proteins responsible for immune cell communication, which makes them a favorable therapeutic for boosting or inhibiting immune responses. Specifically, administration of interleukin-22 (IL-22), which is a cytokine that aids in host cell repair, decreases pneumococcal disease burden as well as viral and gram-negative bacterial pneumonia in animal models of pulmonary infection [23]. Inhibitors of secreted bacterial proteases that cleave protective host cell proteins such as mucins and IgA may also be a promising therapeutic approach [12]. Similar to multivalent vaccines, a combinational therapy may prove to be the most effective treatment course. For instance, a combined therapy targeting the pore-forming toxin pneumolysin and IgA protease could prevent damage while boosting the immune response. Altogether, new therapeutic approaches will diminish our reliance on antibiotics as sole treatment approaches, which is a giant leap forward in preventing the propagation of antibiotic resistance.

## Concluding remarks

Human interventions are training the next generation of pneumococcal superbugs. Natural transformation and high recombination rates in NESp jeopardize treatment outcomes and risk increased propagation of antibiotic resistance. These concerns on selective outgrowth of NESp are relevant to other pathogens such as *Haemophilus influenzae* and *Neisseria meningitidis*, which are undergoing similar ecologically driven population fluctuations based on vaccine and antibiotic selective pressures. As antibiotic resistance emerges and threatens treatment outcomes, we begin to understand the negative consequences of antibiotic usage. Yet the negative consequences of pneumococcal vaccination remain buried by the prevention of severe, invasive disease associated with vaccine serotypes. The future of pneumococcal infections may increasingly involve mucosal surfaces that provide a favored NESp niche. Unfortunately, our current vaccine strategy from target to administration misses the mark at preventing NESp-associated antibiotic-resistant infections. Without intervention of current prevention and treatment measures, the prevalence and severity of NESp-associated diseases will continue to rise.
